# White Matter Changes in Cervical Dystonia Relate to Clinical Effectiveness of Botulinum Toxin Treatment

**DOI:** 10.3389/fneur.2019.00265

**Published:** 2019-04-04

**Authors:** Anne J. Blood, John K. Kuster, Jeff L. Waugh, Jacob M. Levenstein, Trisha J. Multhaupt-Buell, Lewis R. Sudarsky, Hans C. Breiter, Nutan Sharma

**Affiliations:** ^1^Mood and Motor Control Laboratory, Massachusetts General Hospital (MGH), Charlestown, MA, United States; ^2^Laboratory of Neuroimaging and Genetics, Massachusetts General Hospital, Charlestown, MA, United States; ^3^Department of Neurology, Massachusetts General Hospital, Boston, MA, United States; ^4^Department of Psychiatry, Massachusetts General Hospital, Boston, MA, United States; ^5^Martinos Center for Biomedical Imaging, Massachusetts General Hospital, Charlestown, MA, United States; ^6^Department of Psychiatry, Harvard Medical School, Boston, MA, United States; ^7^Division of Child Neurology, Boston Children's Hospital, Boston, MA, United States; ^8^Department of Neurology, Harvard Medical School, Boston, MA, United States; ^9^Department Neurology, Brigham and Women's Hospital, Boston, MA, United States; ^10^Department of Radiology, Massachusetts General Hospital, Boston, MA, United States; ^11^Warren Wright Adolescent Center, Department of Psychiatry and Behavioral Sciences, Northwestern University Feinberg School of Medicine, Chicago, IL, United States

**Keywords:** botulinum toxin, basal ganglia, dystonia, ansa lenticularis, white matter plasticity, diffusion tensor imaging, laterality, repeated measures analyses

## Abstract

In a previous report showing white matter microstructural hemispheric asymmetries medial to the pallidum in focal dystonias, we showed preliminary evidence that this abnormality was reduced 4 weeks after botulinum toxin (BTX) injections. In the current study we report the completed treatment study in a full-size cohort of CD patients (*n* = 14). In addition to showing a shift toward normalization of the hemispheric asymmetry, we evaluated clinical relevance of these findings by relating white matter changes to degree of symptom improvement. We also evaluated whether the magnitude of the white matter asymmetry before treatment was related to severity, laterality, duration of dystonia, and/or number of previous BTX injections. Our results confirm the findings of our preliminary report: we observed significant fractional anisotropy (FA) changes medial to the pallidum 4 weeks after BTX in CD participants that were not observed in controls scanned at the same interval. There was a significant relationship between magnitude of hemispheric asymmetry and dystonia symptom improvement, as measured by percent reduction in dystonia scale scores. There was also a trend toward a relationship between magnitude of pre-injection white matter asymmetry and symptom severity, but not symptom laterality, disorder duration, or number of previous BTX injections. *Post-hoc* analyses suggested the FA changes at least partially reflected changes in pathophysiology, but a dissociation between patient perception of benefit from injections and FA changes suggested the changes did not reflect changes to the primary “driver” of the dystonia. In contrast, there were no changes or group differences in DTI diffusivity measures, suggesting the hemispheric asymmetry in CD does not reflect irreversible white matter tissue loss. These findings support the hypothesis that central nervous system white matter changes are involved in the mechanism by which BTX exerts clinical benefit.

## Introduction

Dystonia is thought to sometimes result from brain plasticity “gone wrong,” with too much of a good thing resulting in pathophysiology of the sensorimotor system (1–3). This is particularly true for dystonias with an “overuse” component, such as musician's dystonia, but may also be true for other dystonias. Moreover, expression of symptoms themselves may help to perpetuate the cycle of aberrant plasticity.

Botulinum toxin (BTX) is frequently used to treat the symptoms of dystonia. BTX reduces synaptic transmission at the neuromuscular junction in a sustained manner over ~2–3 months, and thereby reduces the symptoms of dystonia downstream of presumed pathophysiology in the brain (4). Although BTX appears to show retrograde transport within the primary motor neurons (5), such transport is not known to move above the spinal cord, and BTX does not appear to cross the blood-brain barrier. However, it is believed that changes in brain structure and function following BTX [e.g., (6–11)] are exerted through changes in motor afferent function reflecting the changes in muscle function. Such indirect central effects are thought to facilitate or complement the direct, peripheral effects of BTX in some cases in either an acute or more longitudinal (e.g., plastic) manner, and may temporarily help to break a cycle of excessive function in the sensorimotor circuitry. However, these effects are likely not able to remove the primary driver of excessive central function in dystonia, so once the effects of BTX wear off and muscle function increases again, the central abnormalities are expected in most cases to be reinstated, along with dystonia symptoms.

We previously reported a left/right asymmetry of FA medial to the internal globus pallidus (GPi) in focal dystonia patients, which was not observed in healthy controls. In that same study, we conducted a preliminary assessment of the asymmetry after a subset of these patients (*n* = 4) received BTX treatment to test if symptom reduction led to central changes in this abnormality. We showed that the left/right asymmetry significantly diminished (i.e., partially normalized) 4 weeks after BTX injections in those patients. In the current study, we report the completed, full-scale treatment study in 14 cervical dystonia (CD) patients. Validation of these findings in a full-scale study was particularly important because the preliminary findings led to a hypothesis that a particular subtype of pallidofugal fiber is involved in the pathophysiology of dystonia. Such a hypothesis is only useful and valid if (1) the finding holds up in a full-size cohort and (2) the finding could be in some way linked to expression of dystonic symptoms, such as by relating brain changes to the changes in symptom severity observed after BTX.

The *a priori* regions evaluated in our previous study (12) sought to address the absence of abnormalities in pallidofugal white matter in previous diffusion tensor imaging (DTI) studies of dystonia, which seemed at odds with the fact that this part of the basal ganglia circuitry is thought to be critical to dystonia symptom expression and is the target of deep brain stimulation (DBS) for dystonia (13–15). We hypothesized that negative findings in previous studies reflected the fact that tracts in this region are small and diffuse, and that standard voxelwise approaches to DTI analysis are not targeted enough to these anatomical characteristics; it is well-known that standard voxelwise approaches to DTI analysis are best at detecting changes in large tracts (16). Because of this paucity of empirical data about the basal ganglia in dystonia, our study was designed to be hypothesis-driven with regard to where we expected to see the effects of BTX, but we used a data-driven approach that did not constrain what we expected to see in the findings. We hoped that this might lead to new clues about pathophysiology, rather than to test existing hypotheses about it (a hypothesis-generating approach). We used an *a priori* region of interest (ROI) approach to evaluate the microstructural integrity of pallidofugal fibers by optimizing anatomically defined ROIs for signal specific to these fibers. In the treatment study reported here, we kept our region of evaluation focused on the *a priori* ROIs that showed an abnormality (i.e., a hemispheric asymmetry) in the previous study, to substantially reduce the likelihood of sporadic differences (including false positives) likely to plague repeated measures DTI studies over broad regions (17–19). Note that ROIs were defined and investigated *a priori* both in our previous study and in the current study. The ROIs were defined anatomically and were not based on voxelwise findings from any previous study. Moreover, the abnormality in our prior study was detected independent of any information relating to treatment; they were detected solely by comparing dystonia patient and control values. Because of the way we defined our ROIs in the previous study, we initially used manual segmentation (12). As part of the current study we developed a reliable automated approach to segmenting the *a priori* region of interest (ROI), to eliminate the possibility of human bias in data extraction across individuals and sessions. It was expected this would come at the cost of reduced anatomic accuracy, since nothing replaces the human eye for defining structural landmarks (20, 21), particularly for tracts as small and localized as the pallidofugal fibers, but increased objectivity of the analysis.

While the primary goal of the current study was to advance understanding of dystonia and its treatment, the study is of more broad interest in its implications for adult white matter plasticity. In the years since our 2006 publication, a number of other DTI studies reported evidence for white matter plasticity in adult humans following both, behavioral/cognitive [e.g., (22–27)] and treatment-related (28) interventions. In parallel, there has been extensive discussion of and increasingly compelling evidence for white matter plasticity in the adult brain in the animal literature [e.g., (29–38)]. Although the scope and precise mechanisms of plasticity have not been fully established (36), the literature now suggests that further investigation of white matter changes after BTX is relevant to the evolving field of white matter plasticity.

Our results (1) confirmed reductions in the left/right asymmetry after BTX treatment in the larger CD cohort, (2) showed that these changes were related to the degree of clinical improvement, and (3) showed a trend toward a relationship between pre-treatment left/right asymmetry with pre-treatment symptom severity.

## Materials and Methods

### Participants

#### Participant Inclusion Criteria

Fourteen individuals with a confirmed diagnosis of primary cervical dystonia (CD) (4 males, 10 females, mean age = 52.14 ± 10.41 years), negative for the DYT1 mutation, and having no known history of any other genetic form of dystonia, participated in the study. CD participants were negative for all other neurological diagnoses (including essential tremor), with the exception of one participant who had a history of migraines. Inclusion criteria for this study required that patients be treated with botulinum toxin (BTX) for their dystonia in the timeframe outlined for the imaging protocol (within a week after the first scanning session). This was not, however, a clinical trial; patients received BTX injections as part of their routine treatment and thus injection sites and dosing were selected independent of study parameters.

Fourteen healthy control participants, with no history of or current neurological or psychiatric disorders, and no treatment with neuroactive medications, were matched one-to-one to CD patients by age (within 5 years; average age = 51.64 ± 11.31 years), gender, handedness, and MRI scanner (see Table 1), except for one patient/control pair for which gender was not matched (see Table 1). Controls were confirmed in the clinical screening (see below) to have no evidence of dystonia or other neurological or psychiatric disorders.

**Table 1:** Clinical characteristics of cervical dystonia patients.

**Patient number**	**Age/gender/handedness of patient and (matched control)**	**Regions affected with dystonia and/or dystonic tremor**	**Side(s) affected**	**Tsui scale scores before/after BTX**	**Duration of dystonia**	**No. of prior BTX injections**	**Neuroactive medications**
1^*^	55/F/left (ctrl:52/F/left)	Neck, with dystonic tremor	both, with left head tilt	8/4	35 years	12 prior inj	Eletriptan (as needed for migraine, but not at time of scans)
2^*^^†^	59/M/right (ctrl:59/M/right)	Neck	Both, with right head tilt	11/6	34 years	18 prior inj	None
3^*^	35/F/right (ctrl:31/M/right)	Neck, trunk, craniofacial	Both, with left head tilt, right rotation, right craniofacial	^‡^BFM: 18/8; Tsui: 4/2	13 years	10 prior inj	None
4^*^	57/F/right (ctrl:59/F/right)	Neck, with dystonic tremor	both^*^, with left head tilt, retrocollis	7/2	41 years	11 prior inj	None
5	59/M/right (ctrl:60/M/right)	Neck	Right tilt	8/2	20 years	25 prior inj	None
6	46/M/right (ctrl:45/M/right)	Neck	Right tilt, shoulder elev	4/3	7 years	9 prior inj	Tramadol
7	50/F/right (ctrl:49/F/right)	Neck	Right rotation	6/6	5 months	No	Clonazepam
8	37/M/right (ctrl:37/M/right)	Neck	both, with left tilt, right lateral shift	3/6	3–4 years	1 prior inj	Clonazepam 4 months prior to scan but none at time of scan
9	38/F/right (ctrl:41/F/right)	Neck	Both, with right rotation, left tilt	6/4	8 or 9 years	2 prior inj	Diazepam as needed, last dose 3-4 wks before scan
10	52/F/right (ctrl:51/F/right)	Neck	right rotation, head tilt	11/7	6 years	1 prior inj	Hydrocodone, diazepam
11	64/F/right (ctrl:64/F/right)	Neck	Left tilt, shoulder elevation/tremor	7/5	10–15 years	No	Venlafaxine
12	49/F/right (ctrl:47/F/right)	Neck	Right rotation	6/5	1 year	1 prior inj	Tolterodine
13	70/F/right (ctrl:74/F/right)	Neck	Right rotation	2/2	4 years	5 prior inj	None
14	59/F/right (ctrl:54/F/right)	Neck	Right head tilt	4/2	3 years	1 prior inj	Lorazepam

*BFM, Burke Fahn Marsden dystonia rating scale*.

*Tsui, Tsui rating scale for cervical dystonia*.

* *Subjects included in 2006 publication using different analyses*.

† *This subject was included in the 2006 publication, but returned for a second set of scans on the new magnet for the current study; only new scan/magnet data was used for this subject in the current study, and a new matched control was recruited and scanned on the new magnet*.

‡ *BFM scale scores are included in addition to Tsui since this patient had multifocal dystonia (see section Materials and Methods for use of the scales)*.

#### Clinical Screening

Immediately prior to each scan session, all participants completed a clinical history (including past and current medications) and a clinical assessment, including a standardized motor exam used to rule out motor abnormalities in the control group and to complete dystonia scales in both groups (Burke Fahn Marsden [BFM], Tsui, and TWSTRS dystonia severity scales). A description of the motor exam protocol can be found in Supplementary Methods (Supplementary Material Table S1). Motor exam assessment was completed by a movement disorders physician (N. Sharma). Raw data for the Tsui dystonia severity scale and site/dosing information for BTX injections is included as Supplementary Clinical Data (Supplementary Material Data Sheet S1). All control subjects scored zero on the dystonia scales. All clinical screening was completed blind to information about imaging findings in patients and, similarly, imaging was conducted blind to information about clinical screening information.

The Tsui cervical dystonia scale was used as the primary form of evaluation and quantification of response to BTX; we used the total Tsui score as our main measure quantifying dystonia severity before vs. after treatment (i.e., treatment effectiveness). We also used subsections of this scale to evaluate positional changes in our *post-hoc* analyses. The Tsui scale was used because several patients at the beginning of the study had not been evaluated with the TWSTRS at one or both scanning sessions; in addition, we agreed with a previous review of cervical dystonia scales for use in the evaluation of BTX that the TWSTRS includes a number of components that capture more qualitative than quantitative aspects of dystonia (e.g., sensory tricks, range of motion, etc.) that may affect the burden of the disease but do not necessarily reflect quantity of pathophysiology or response to treatment (39). We further agreed with this review that the duration factor in the TWSTRS contributes an unjustified weight, particularly due to its weighting by a factor of 2 (39). Most subjects in this study scored 10 on this factor and this led to underweighting of the severity of dystonic positioning. However, we provide statistics and Supplementary Figures S1, S3) showing the two main regression analyses for those patients with TWSTRS scores, for comparison; individual total scores for TWSTRS are also included in Supplementary Clinical Data (Supplementary Material Data Sheet S1). Further details about quantification and use of dystonia scales for evaluation with imaging analyses are described below in the Data Analyses section.

In addition, we asked the patients a set of questions about their own perception of symptoms and responses to BTX and used the answer to one of these questions in our analyses. Specifically we simply asked whether they felt any benefit from the injections (with answers being segregated into “at least some benefit” or “no benefit”). Questionnaire data was available for 11 of the 14 patients.

Table 1 characterizes the demographics and clinical profiles for each individual with CD, including duration and severity of the disorder (as measured by the BFM and Tsui severity scales) and current medication status. We also aimed to include patients with a range in number of previous BTX injections to rule out a relationship between number of injections and asymmetry (i.e., is there a possibility the asymmetry is a result of receiving this treatment over time, rather than a marker for dystonia; see Table 1). No oral medications were withheld for the purposes of the study; patients listed as “no” for oral medications in Table 1 were patients who were treated sufficiently well by BTX to not require oral medications for dystonia. Any patients who were on oral medications had been stabilized on these medications before being enrolled in the study (no changes to medications or dosing for at least 3 months prior to the first scan session), and no changes were made to any of these medications between scanning sessions.

### Imaging Acquisition

All participants were scanned twice, using the same procedures and imaging sequences in each session. For the first session, all CD patients were scanned within the week before their next scheduled BTX injection. Thus, for patients who had received previous BTX injections, this first scanning session coincided with the end of a BTX treatment period so symptoms were at their worst, and effects of BTX minimal. For the second session, CD patients were scanned 4 weeks after their injections, resulting in a 4–5 week interval between scan sessions. Healthy controls were scanned at the same interval (4–5 weeks apart). Although it was not expected that controls would experience any brain changes over this time period (as they had no intervention), there is little short-interval, repeated measures DTI data reported in the literature, and thus little is known about how much normal variation there is due to experimental factor variability or physiological fluctuations across sessions (40). For example, head motion can affect diffusion images and lead to spurious group differences (18). It was thus important to provide healthy control test-retest data (as well as motion data for patients; see below) as a negative control condition. Participants were scanned on a 3.0 Tesla Siemens Tim Trio magnet system (Siemens AG, Medical Solutions, Erlangen, Germany), except for three of the patients and matched controls included in the 2006 publication who were scanned on a 3.0 Tesla Siemens Allegra magnet. One patient previously included in the 2006 publication returned for a new pair of scans on the Trio magnet; only these new scans were used in the current study with a new matched control also scanned on the Trio magnet. One-to-one matched patient-control pairs were always scanned with the same magnet, and the same magnet was always used across sessions within-subject such that magnet identity had minimal impact on our analyses.

During each imaging session we acquired one high-resolution whole brain DTI sequence he following sequence parameters: repetition time (TR) = 24 s; echo time (TE) = 81 ms; slice thickness = 2 mm isotropic, 60 slices total, acquisition matrix 128 × 128 [256 × 256 mm field of view (FOV)], six averages, 60 non-colinear directions, with *b*-value = 700 s/mm^2^, and one image with *b*-value = 0 s/mm^2^. DTI scans were acquired using auto-align software (41) so that brain image slice orientation was the same across participants and sessions.

### Data Analyses

All image analysis was performed using the Diffusion Toolbox (FDT v2.0) from The Oxford Centre for Functional Magnetic Resonance Imaging of the Brain (FMRIB) software (http://fsl.fmrib.ox.ac.uk/fsl/fslwiki/) version 4.1.4, with standard analysis parameters. Raw data used in imaging analyses (i.e., data extracted from ROIs) is included as Supplementary Imaging Data files for FA, MD, RD, and AD values (Supplementary Material Data Sheets S2–S5).

#### Preprocessing

The initial data preprocessing for each subject included image orientation, removal of non-brain tissue (BET), and correction for head motion and eddy current distortions. DTIFIT reconstruction of diffusion tensors was applied at each voxel to create 3D images at the same matrix size and resolution as the original diffusion images, including fractional anisotropy (FA), mean diffusivity (MD), and eigenvalue (L1, L2, L3) maps for each subject. MD and eigenvalue maps were used to evaluate diffusivity, as described below.

#### Registration Procedures

FA, MD, and eigenvalue maps were registered into MNI standard space using the FSL non-linear transform (i.e., FNIRT), registering to the FSL DTI template FA map (FMRIB58_FA_1 mm_brain) using FSL standard default parameters for DTI registration (https://fsl.fmrib.ox.ac.uk/fsl/fslwiki/FDT/UserGuide#Registration_within_FDT).

#### FA Region of Interest (ROI) Automated Extraction Methods

**Figure 1:**
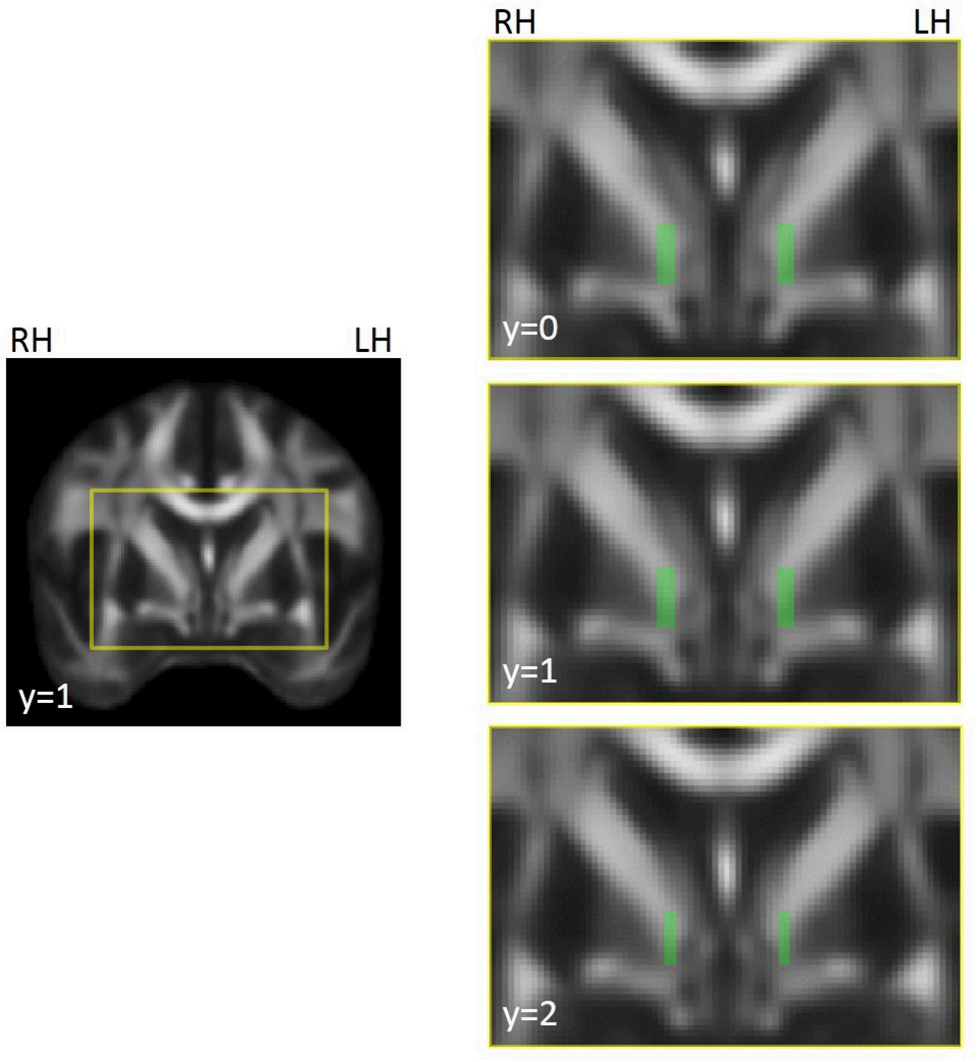
Illustration of ROI template for FA extraction in left and right hemispheres, coinciding with the location of the region showing left/right asymmetries in focal dystonia patients (12). The voxels highlighted in green in the images on the right were compiled into a mask across slices, separately for the left and right hemispheres. The masks were used to extract FA in underlying voxels from the FA maps for each participant and session. ROIs were created on the FSL MNI FA template (FMRIB58_FA_1 mm) and were used to extract data from individual FA maps that had been registered to this template. The yellow box in the left hand image shows the field of view for the images on the right. MNI coordinates are indicated for each slice. RH, right hemisphere; LH, left hemisphere.

For automated extraction of FA in white matter medial to the GPi, we created a pair of left and right template ROIs on the FSL MNI FA brain (Figure 1) that were as anatomically similar as possible to the manually drawn, native-space ROIs used previously (12). We created these ROIs only for the three slices that previously showed group differences. Note that ROIs were not selected in the current study to show patient/control differences; they were selected as known abnormalities (identified in our previous study) in order to evaluate treatment effect on dystonia-relevant circuitry. Note, also, that both manually drawn and automated ROIs were defined *a priori* and anatomically and were therefore defined independent of previous findings (i.e., there was no circularity in the selection of which voxels were extracted from a given slice). This region anatomically coincided with the ansa lenticularis, one of the main efferent projections of the GPi (42). Template ROIs were left/right symmetrical in location, shape, and volume, and similar in size/shape to the hand drawn ROIs used previously (12). Using the FMRIB program *fslmeants*, template ROIs were used to extract FA from corresponding voxels in each subject's MNI-registered FA map and FA was averaged across the voxels of each ROI for left and right hemispheres.

#### Comparison of FA Asymmetries Before vs. After BTX

For each participant and session, the average FA for the right hemisphere ROI was subtracted from the average FA for the left hemisphere ROI, as in our previous study, to calculate a left/right “asymmetry” value. Our preliminary study had not been aimed at detecting an asymmetry but our data-driven approach indicated that this was the most dominant finding across groups. Given the small sample size it was not clear if it reflected an abnormality lateralized to one side or if it was the asymmetry itself that was the issue. So as we completed the full-scale study we continued to use left/right difference as our primary measure. Given the complexity of anatomy in this region, this approach of calculating relative left/right differences serves a further methodological benefit of reducing between-subject noise. In other words, this approach served to normalize data across subjects, but was not aimed *a priori* at identifying a specific biological etiology. We include *post-hoc* analyses of data from individual hemispheres to determine if both hemispheres were abnormal (in opposite directions) or if the results simply reflecting a finding lateralized to one hemisphere. Similarly, we asked how FA changes in each hemisphere after treatment related to changes in dystonia severity, to determine if asymmetry changes with treatment were driven by effects in a single hemisphere.

As in our previous study (12), we used a repeated measures ANOVA with mixed models (xlstat, 2017) to test if there was a group × time (i.e., first vs. second scan session) interaction on the left/right asymmetry. That is, did the FA asymmetry change based on scan session number, and was this effect relevant to both groups or just to the group receiving BTX injections (i.e., patients)? Sign information was maintained during this comparison such that the direction of left/right asymmetry (left < right vs. left > right) was taken into account as part of the comparison. As this was our only *a priori* hypothesis tested here, we used a threshold of *p* < 0.05 for significance. We calculated the average relative difference (i.e., change) in asymmetry values across sessions for participants in each group for graphic visualization of differences between groups across time.

#### Diffusivity (MD, RD, AD) Region of Interest (ROI) Automated Extraction Methods

As a complementary analysis to aid in interpretation of our *a priori* hypothesized changes in FA asymmetry, we also evaluated three diffusivity measures—mean diffusivity (MD), axial diffusivity (AD), and radial diffusivity (RD)—in the region where FA was evaluated. Diffusivity measures reflect the degree of water movement for all orientations (MD) or in a particular orientation (AD and RD), as compared with the imbalance in diffusion directions measured by FA. MD was extracted from MD maps generated as part of DTIFIT (see above). The first eigenvalue map (L1) was used for the AD measure (43) and the second and third eigenvalue maps (L2 and L3) were used for the RD measure, with the following computation: RD = (L2+L3)/2 (43). We extracted these values using the same procedures as for FA values described above.

#### Comparison of Diffusivity (MD, RD, AD) Asymmetries Before vs. After BTX

We compared MD, AD, and RD asymmetry changes across time in the same manner as described above for FA, using a mixed models repeated measures. In addition, to test whether FA differences across groups might reflect pathology/axonal loss in the CD group, we compared diffusivity measures within each hemisphere across CD and controls for the pre-injection scan session, using two-sample *t*-tests. Because we evaluated two comparisons for each non-independent diffusivity modality, we used a Bonferroni correction for two comparisons each, or a significance threshold of *p* < 0.05/2 = 0.025. Given the significance threshold for our primary *a priori* hypothesis using FA measures was *p* < 0.05, we also took into consideration any contrast that met *p* < 0.05 as a trend toward significance. Finally, we used linear regressions to evaluate whether there was a relationship between FA and diffusivity asymmetries within the CD group before BTX; we therefore corrected for three comparisons ((1) MD vs. FA, (2) AD vs. FA, and (3) RD vs. FA) using the bonferroni method, for a significance threshold of *p* < 0.05/3 = 0.0167).

#### Relationship of FA Left/Right Asymmetry Changes to Clinical Improvement Before vs. After BTX

We performed a *post-hoc* linear regression analysis between (1) percent reduction in dystonia scale score for patients before vs. after BTX and (2) the change in left/right FA asymmetry before vs. after BTX (including directional information). Percent scale score reduction was calculated using the following algorithm: (score before BTX – score after BTX)/score before BTX. One patient exhibited multifocal dystonia that affected trunk and face in addition to neck and received BTX injections in all affected regions. Since we were interested in white matter changes relating to all regions injected with BTX (we believe that the asymmetry reflects net impact of dystonia symptoms across the body, see similar effects in focal hand dystonia in (12)), we calculated the percent score reduction using BFM scores for this participant to capture all affected/injected regions. In this case it was legitimate to use different scales since scales were normalized by calculating a within-subject percent change and were thus unit-less, normalized covariates. Note that the percent reduction in Tsui and BFM scores was nearly identical in this patient (% reduction of 50% for the Tsui score and 56% for the BFM score), indicating that cervical and non-cervical symptoms were similarly responsive to BTX. Since we were uncertain if the FA asymmetry itself was relevant to understanding pathophysiology or if the left/right difference simply reflected a lateralized finding, we computed an evaluation of this regression analysis separately for left and right hemispheres.

Dystonia scales are a coarse and imperfect way of evaluating these effects since they only capture the net degree to which BTX has improved head position; nevertheless, they are the best quantitative measure we have at this time to make this evaluation, so this was the measure used to evaluate symptom improvement in the current study. Given that we conducted a total of three regressions with clinical measures (including the relationship of dystonia duration and severity to asymmetry, below), we corrected regression *p*-values for three comparisons using the bonferroni method such that significance was established at *p* < 0.05/3, or 0.0166.

One specific reason, from a study design perspective, that dystonia scales were imperfect for quantifying symptom reduction here, is that we deliberately included CD participants who had received few to no previous BTX injections so we could evaluate whether the injections themselves might somehow be associated with development of the abnormal asymmetry. However, it often takes several rounds of BTX injections before the site and dose are optimized, and dystonia can be unmasked in uninjected muscles. When this happens, dystonia scales may not improve for those individuals, even though the total amount of dystonic activity across the neck and underlying brain pathophysiology are reduced. Thus, for individuals whose scale scores do not improve, scale scores would not accurately reflect underlying biology, and therefore should not be included in analyses evaluating relationships between scale change and biology. In this study we therefore computed regressions between dystonia scale scores and change in left/right FA asymmetry only for patients who experienced a net improvement in their scale scores (11 of 14 patients).

#### Relationship of Hemispheric Asymmetries in CD to Symptom Duration and Pre-injection Severity

We used a linear regression to evaluate the relationship between left/right FA asymmetries before BTX and dystonia duration in years. We also used a linear regression to evaluate the relationship between pre-injection left/right FA asymmetry and pre-injection dystonia severity to investigate whether left/right asymmetries might be relevant to clinical history. We calculated the duration measure relative to the time of first reported symptoms, rather than time of diagnosis, given there tends to be a broad range of time to diagnosis across patients. The patient exhibiting multifocal dystonia that affected trunk and face in addition to neck was excluded from the pre-injection left/right FA asymmetry and pre-injection dystonia severity regression analysis, since the absolute scores for BFM and Tsui (as compared with unit-less relative change in score within subject) could not be combined as covariates, and it would have been inaccurate to relate the Tsui score alone to the asymmetry. Note that this patient with multifocal dystonia was included in all other analyses in this study, including our *a priori* analysis evaluating change in FA asymmetry across scan sessions, and the regression of this change with reduction in scale score. We corrected *p*-values for three total FA evaluations against clinical features (including the clinical improvement regression described above) using the Bonferroni method such that *p* < 0.05/3, or 0.0166.

#### Evaluation of FA Asymmetry in Relation to Laterality of Dystonia

Given that our previous study showed left FA lower than right FA in all focal dystonia patients with varying symptom laterality, we predicted that the direction of microstructural asymmetry in the current study would not relate to anatomical laterality of symptoms. Although we are not yet completely certain why this was the case, we believe it is most likely related to the laterality of motor function subsystems, reflecting the laterality of the specific motor subsystem(s) affected in dystonia (44, 45). We evaluated this relationship as a negative control analysis, using information about symptom laterality based on the number of BTX units injected on the left vs. right side of the neck following the “before BTX” scan (the hemispheric botulinum ratio (46)). Specifically, we conducted linear regression analyses to evaluate the relationship between (1) left/right FA asymmetry in the “before” scan and (2) degree and direction of asymmetry of BTX units injected into neck muscles following the “before” scan. BTX asymmetry was calculated as left side units minus right side units divided by total units, such that a complete left dominance (i.e., left only) would be denoted by +1, complete right dominance (i.e., right only) by −1, and equal left/right units as zero. Because the sternocleidomastoid (SCM) muscle turns the head in the direction opposite to its anatomical laterality, we ran the analysis two ways, first classifying the SCM by anatomical laterality (ipsilateral) and then a second time, classifying it by directional laterality (contralateral). Since this was a negative control analysis we required *p* > 0.1 to confirm our hypothesis that there would not be a relationship between these measures.

As a complementary analysis we asked the same question about laterality using a different approach: Specifically, we classified patients as left- or right-dominant based on the direction of head tilt and/or shoulder elevation. While the presence of head rotation led to bilateral muscle involvement in most patients, all patients lateralized to the left or right regarding head tilt and/or shoulder elevation and were stratified by this factor for this analysis. We felt this was justified because, with the exception of two patients, all patients with head rotation were lateralized in the same direction (right rotation). One of the patients who showed a left rotation was left handed (the only left-handed patient in this study), and the other, who was right-handed, had originally presented with a right rotation that had “migrated” over the years after numerous BTX injections. For this analysis we excluded patients who presented with head rotation only (*n* = 10). Using the predominant symptom laterality for each patient, we evaluated if there were differences in FA asymmetry improvement after treatment for left vs. right-lateralized symptoms. Only patients with a lateralized tilt and/or shoulder elevation were included in this laterality analysis; patients with head rotation only were excluded from this analysis. For this *post-hoc* analysis we used a two-sample *t*-test to compare FA changes (with treatment) across left- and right-lateralized patients. Since this was a negative control analysis we required *p* > 0.1 to confirm our hypothesis that there would not be a relationship between these measures. In addition, we evaluated how patient perception of benefit from treatment (“at least some benefit” vs. “no benefit”) related to this classification of laterality, making qualitative observations how perception of benefit aligned or did not align with symptom laterality. For this evaluation we included only patients who had both a head tilt and/or shoulder elevation and a response to the “benefit” question (*n* = 8).

#### Post-hoc Analyses to Dissociate Pathophysiology From Compensation, and to Dissociate Different Aspects of Pathophysiology

While symptom severity and compensation for or adaptation to the disability caused by symptoms often go hand in hand, we aimed to find ways that compensation/adaptation may have been dissociated from pathophysiology to test the likelihood that imaging findings related to pathophysiology rather than to some other factor. We noted that in our cohort there were patients that showed normalization of FA asymmetry and felt they benefited from the injections, but did not show improvement in head position (see Supplementary Clinical Data in Supplementary Material Data Sheet S1). While there were only three of these patients, these values could provide at least preliminary evidence whether FA changes reflected less demand for compensation/adaptation due solely to head position. Specifically, we asked if the relative magnitude of postural effort required to counteract head deviation before treatment correlated with FA measures before treatment (FA asymmetry, left hemisphere FA, and right hemisphere FA) less robustly than the main regression against total severity ratings. We estimated this effort value by adding up the head deviation scores in the Tsui dystonia scales (scores for rotation, head tilt, antero- or retrocollis, and shoulder elevation, independent of direction). We also asked if reduced (or increased) effort values after treatment related to changes in these FA measures less robustly than total severity ratings. We used linear regressions to evaluate these relationships.

We next asked a more complex question as an exploratory analysis: did patient perception of benefit from injections (“at least some benefit” or “no benefit”) relate to FA changes after treatment? We asked this both because perceived benefit should influence more psychological (as opposed to mechanical) components of disability distinct from pathophysiology itself, and because this question also gets at how directly our effects localize to the “driver” of dystonia, as opposed to simply its expression. As noted above, we noted that there was not a strict relationship between perceived benefit and positional improvement in our raw data (see Supplementary Clinical Data in Supplementary Material Data Sheet S1), suggesting that positional correction may not accurately reflect what the patient experiences with regard to treatment benefit. For this second comparison we used a *t*-test to compare FA asymmetry changes for patients who experienced “at least some benefit” vs. those who reported “no benefit.”

#### Evaluation of FA Asymmetry in Relation to Number of Previous BTX Injections (Control Analysis)

We aimed to include patients with a range in number of previous sets of BTX injections to rule out a relationship between number of previous injections and asymmetry (i.e., is there a possibility the asymmetry is a result of receiving this treatment over time, rather than a marker for dystonia?). We used a linear regression to evaluate the relationship between number of times a patient previously received BTX injections and the left/right difference in FA values for our *a priori* ROIs at the pre-injection (first) scan session. Since this was a negative control analysis we required *p* > 0.1 to confirm our hypothesis that there would not be a relationship between these measures.

#### Assurance That Head Position or Movement did Not Contribute to Effects (Control Analysis)

FA does not convey information about absolute direction of water diffusion. This measure conveys information only about the relative homogeneity or inhomogeneity of diffusion directions in a given voxel (47). Nevertheless, in our previous DTI study (12) we used rigorous procedures to be certain that possible deviations of patient head position did not influence DTI left/right asymmetry measures, and it was determined that head position had no influence on these measures. Two procedures were used in the current study: The first was the use of autoalign software (41) to ensure that slices were acquired in the same orientation across subjects and scanning sessions within a subject (aligned with the AC/PC line, independent of head position). Using this software, images are acquired relative to the head position itself, not to the absolute position of the head in the scanner, and thus head position has no bearing on data acquired. The second was to evaluate head movement (a) across groups and (b) within group, across session, for the CD group. Head movement was calculated using the output of the preprocessing motion correction step. This step produces measures of rotation and translation, and computes a net movement vector (which includes directional information) for each acquisition frame of the DTI sequence. We used the average of this net movement vector (across the sequence) in the following analyses: (1) a *t*-test to compare movement for CD vs. controls and (2) a linear regression in CD participants to evaluate the relationship between movement difference across scan sessions and difference in left/right FA asymmetry across sessions.

## Results

Treatment with BTX injections was associated with reduced left/right FA asymmetry, and magnitude of left/right asymmetry reduction was related to clinical effectiveness of treatment. In contrast, diffusivity measures (which reflect how far water diffuses, rather than the direction of diffusion) showed neither group differences nor changes across scan sessions. FA changes in patients were not related to duration of dystonia, laterality of symptoms, previous number of BTX injections, or head motion parameters.

### Comparison of FA Asymmetries Before vs. After BTX

**Figure 2:**
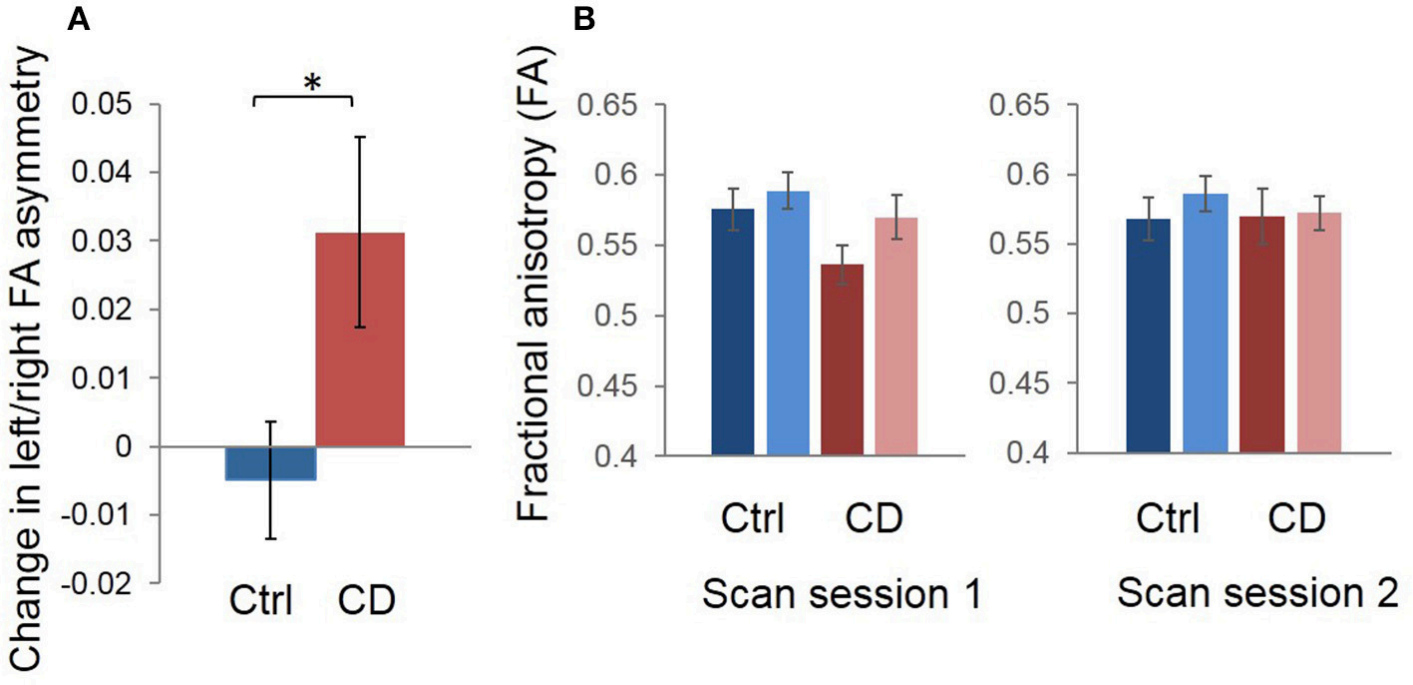
Group mean changes in left/right FA asymmetry in our region of interest, for scan session 1 vs. scan session 2 (i.e., distance between signed left/right asymmetry values for the two sessions). **(A)** The control mean change is shown in blue and CD mean change is shown in red. Error bars represent the standard error of the mean; asterisk denotes statistical significance. We calculated the average relative difference (i.e., change) in asymmetry values across sessions for participants in each group for graphic visualization of differences between groups across time. The sign of asymmetry and difference values was maintained during this process such that the difference calculations measured the distance between and direction of change in asymmetry values. Thus, a positive change reflected a reduction in left/right asymmetry, with left FA increasing and/or right FA decreasing, while a negative change reflected an increase in left/right asymmetry, with left FA decreasing and/or right FA increasing. **(B)** Mean FA values for left and right hemispheres for the first vs. second scan sessions are also shown to complement the left/right asymmetry session difference data in **(A)**. It appears that, on average, the asymmetry change shown in **(A)** in patients was driven primarily by a normalization of left FA values after treatment. Blue = control; Red = patient.

There was a significant group (CD vs. control) x time (before vs. after BTX) interaction on FA asymmetry (*F* = 4.94; *p* = 0.035). That is, across-session FA asymmetry changes differed between treated patients and untreated controls. Average within-subject changes in FA asymmetry are shown in Figure 2A for the two groups: mean shift in left/right asymmetry from the first to the second scan session was 0.0313 ± 0.0139 (standard error of the mean [SEM]) for CD patients with treatment, and −0.00490 ± 0.00854 (SEM) for healthy controls without treatment. As in our previous study (12), average direction of asymmetry change for patients from before to after treatment was positive (i.e., with left hemisphere FA increasing relative to the right).

Group averages for left and right hemispheres suggested that the main effects were driven by lower FA in the left hemisphere in patients before treatment, and that FA increased in this hemisphere after treatment, with right FA appearing normal before treatment and unchanged afterwards (Figure 2B). However, these are only qualitative, group observations and there was heterogeneity across patients with regard to whether left FA increased or right FA decreased (or both) after treatment.

### Comparison of Diffusivity (MD, RD, AD) Asymmetries Before vs. After BTX

There were no differences in mean diffusivity (MD), radial diffusivity (RD), or axial diffusivity (AD) measures between CD and controls pre-injection, and no group x time interaction for diffusivity asymmetry measures (all *p* > 0.05, uncorrected). There was also no relationship between FA and diffusivity left/right asymmetry measures in the CD group before BTX (all *p* > 0.05, uncorrected).

### Relationship of FA Changes to Clinical Improvement Before vs. After BTX

**Figure 3:**
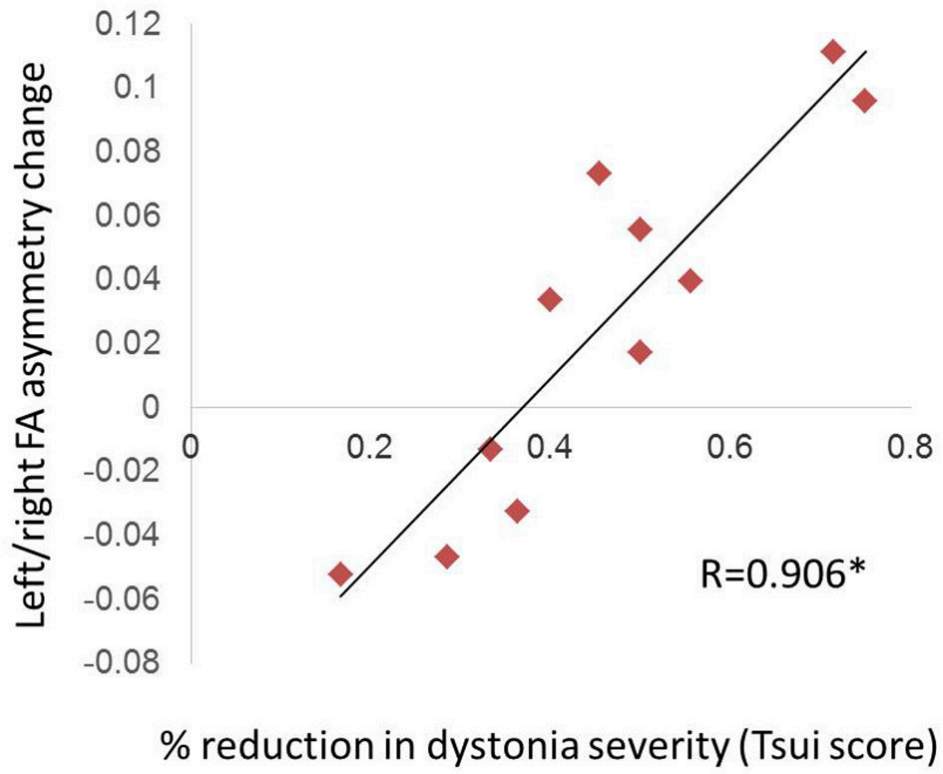
Relationship of FA asymmetry changes to clinical improvement in CD. The change in left/right asymmetry before vs. after BTX correlated with the percent reduction in Tsui dystonia scale severity (significant [denoted by asterisk with *R*-value], at *p* = 0.0001). We computed this correlation only for patients who experienced a net improvement in their scale scores (11 of 14 patients; see section Materials and Methods).

For the 11 CD patients who showed a net improvement in dystonia symptoms after BTX, there was a significant positive relationship between Tsui dystonia scale score reduction after BTX and change in FA asymmetry values (*R* = 0.906, *p* = 0.0001; Figure 3); that is, greater clinical improvement was associated with greater positive change in asymmetry measures after treatment. Similar qualitative relationships were shown using the TWSTRS scale, but were not as robust as for the Tsui scale (Figure S1; *R* = 0.719, *p* = 0.107).

When the above relationship was evaluated separately against FA changes in left and right hemispheres, the findings suggested that reductions in asymmetry with treatment were driven by both left FA increases and right FA decreases, although left hemisphere changes appeared more consistent and tightly correlated with symptom severity changes (Figure S2; For left hemisphere: *R* = 0.739, *p* = 0.00940; For right hemisphere: *R* = 0.612, *p* = 0.0454). However, correlations with the asymmetry itself (as reported in the paragraph above) were more robust than with either individual hemisphere.

### Relationship of Hemispheric Asymmetries in CD to Symptom Duration and Pre-injection Severity

**Figure 4:**
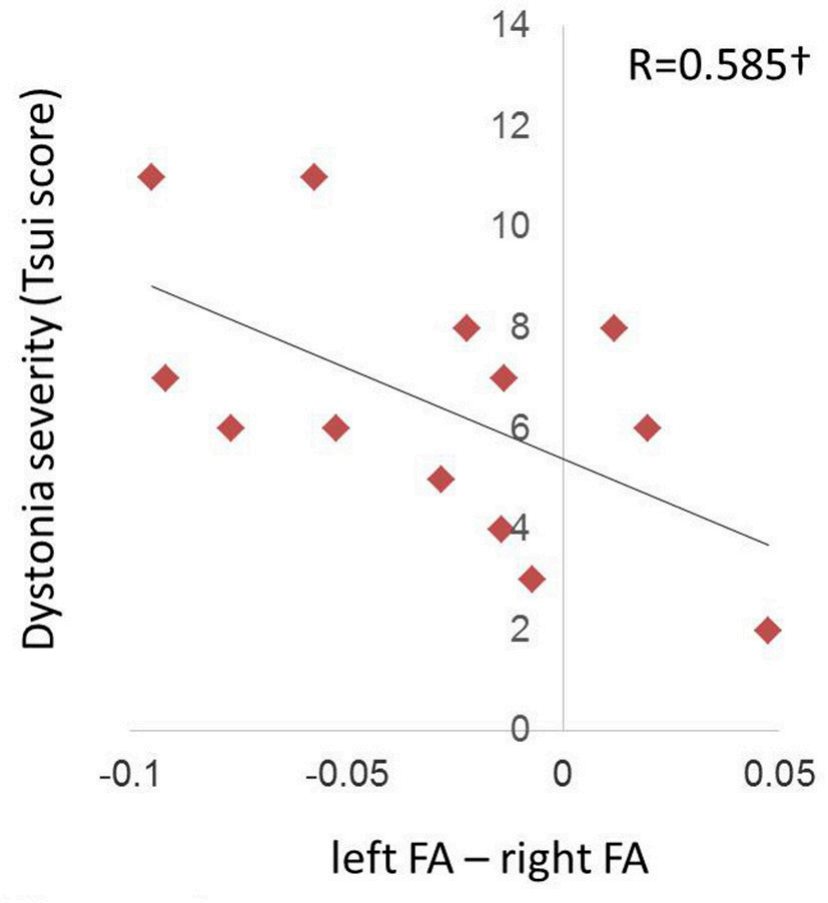
Relationship of FA hemispheric asymmetries in CD to symptom severity at the pre-injection (“before BTX”) scan. The left/right FA asymmetry (left minus right, including directional information) before BTX showed a trend (denoted by ^†^) toward a relationship with dystonia severity as measured by the Tsui scale for cervical dystonia (*p* = 0.0358). Higher Tsui scores were associated with left hemisphere FA being more reduced relative to right FA.

At the “before BTX” scan session, left/right FA asymmetry showed trend toward a significant relationship with Tsui dystonia severity scores (*R* = 0.573, *p* = 0.0407; Figure 4), but there was no relationship to duration of the disorder (*R* = 0.397, *p* = 0.160). Similar qualitative relationships were shown using the TWSTRS scale, but were not as robust as for the Tsui scale (Figure S3; *R* = 0.545, *p* = 0.0669).

### Evaluation of FA Asymmetry in Relation to Laterality of Dystonia

**Figure 5:**
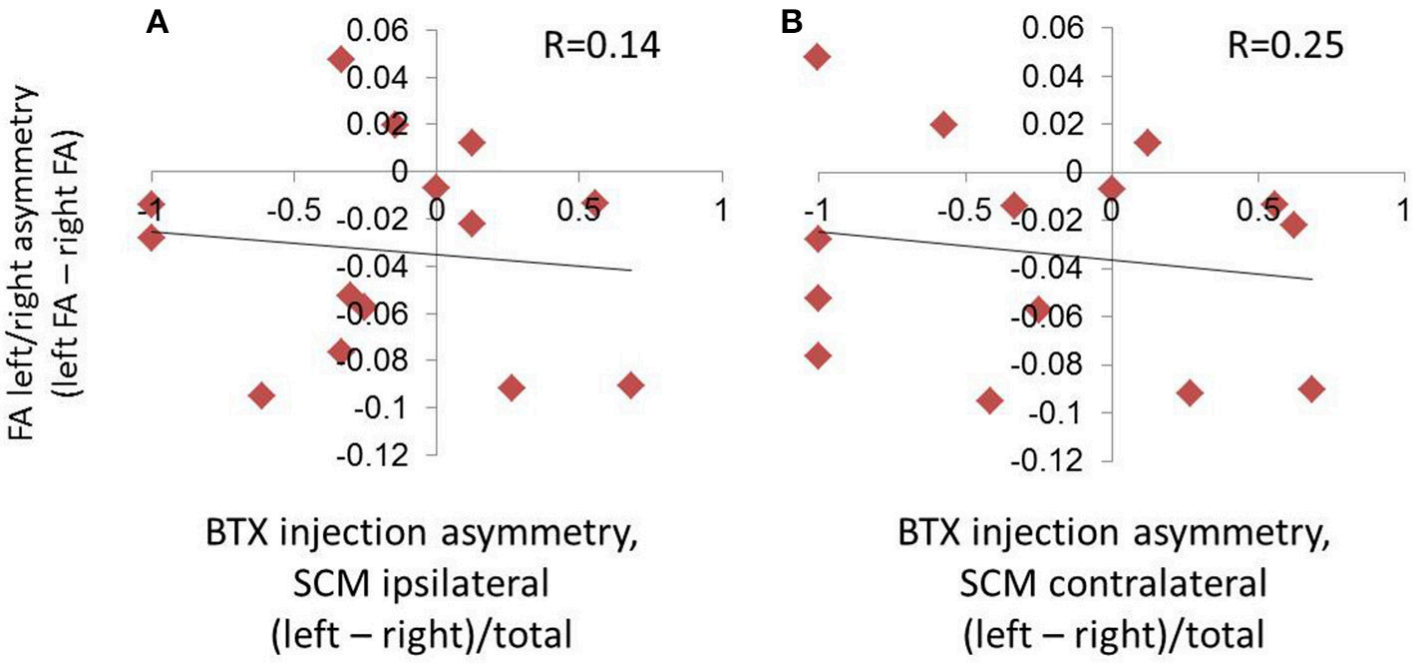
Absence of relationship between hemispheric asymmetries in FA and symptom laterality, as evaluated by the laterality of BTX injections. Symptom laterality was quantified using the proportion of left vs. right BTX units injected (calculated as left units minus right units divided by total BTX units). Given that the sternocleidomastoid (SCM) muscle produces movement contralateral to its anatomical location, we assessed laterality using two parallel methods: in **(A)**, we used the side of the muscle location, and in **(B)**, we used the direction of movement produced by contraction of a given muscle. This meant that the SCM was classified ipsilaterally in **(A)**, and contralaterally in **(B)**.

There was no relationship between laterality of BTX units and laterality of FA measures (*p* = 0.62 when SCM was classified ipsilaterally [anatomical side, Figure 5A]; *p* = 0.39 when SCM was classified contralaterally [direction of movement produced by muscle, Figure 5B]).

There were no differences in FA asymmetry changes with treatment for left vs. right-lateralized symptoms when head tilt/shoulder elevation was used as the primary classifier of laterality (*t* = 0.220, *p* = 0.831). In contrast, there was 100% alignment between head tilt/shoulder elevation laterality and whether patients perceived a benefit, for patients that had both measures (head tilt/shoulder elevation AND a response to the “benefit” question). All patients with left-lateralized symptoms (i.e., relating to head tilt and/or shoulder elevation) reported feeling at least some benefit from treatment, while all of those with right-lateralized symptoms reported feeling no benefit (see Supplementary Clinical Data in Supplementary Material Data Sheet S1).

### *Post-hoc* Analyses to Dissociate Pathophysiology From Compensation

There were no significant relationships between effort required to counteract head deviation and FA measures (i.e., the FA asymmetry, and left and right hemisphere FA) before treatment, nor were changes in effort required to counteract head deviation related to FA changes (i.e., changes in the FA asymmetry, and in left and right hemisphere FA) after treatment (*p* > 0.10 for all of these relationships; Figures S4, S5). There were no differences in FA changes with treatment for those who did vs. did not perceive benefit from treatment (*t* = 0.682, *p* = 0.513).

### Evaluation of FA Asymmetry in Relation to Number of Previous BTX Injections (Control Analysis)

**Figure 6:**
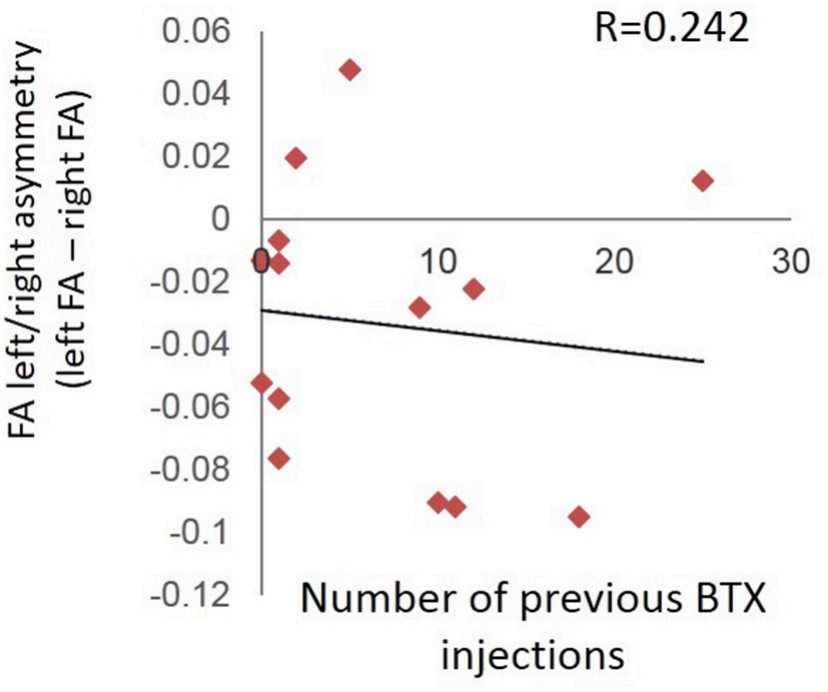
Absence of relationship between number of times a patient previously received BTX injections and the left/right difference in FA values for the pre-injection (first) scan session.

There was no relationship between number of times a patient previously received BTX injections and the left/right difference in FA values for our *a priori* ROIs at the pre-injection (first) scan session (*R* = 0.242; *p* = 0.404) (Figure 6).

### Assurance That Head Position or Movement did Not Contribute to Effects (Control Analysis)

There were no group differences in head movement across CD and control groups before BTX (*t* = 0.225; *p* = 0.824; mean displacement difference for CD = 0.0190, mean displacement difference for control = 0.0329), nor was there a relationship between head movement differences across sessions and left/right FA asymmetry differences across sessions in CD patients (*R* = 0.0439, *p* = 0.882).

## Discussion

In this study we detected changes in the left/right asymmetry of white matter microstructure medial to the pallidum after BTX injections in individuals with CD. There was a strong relationship between the degree of asymmetry change and clinical effectiveness of the treatment. We showed that the direction of left/right symptom asymmetry was not related to the direction of left/right FA asymmetry, while dystonic severity was weakly (trend-level) related to FA asymmetry magnitude. There was no relationship between the asymmetry and number of previous BTX injections, suggesting the asymmetry did not emerge as a result of repeated injections.

A number of previous studies have shown central changes after BTX treatment in dystonia [e.g., (6–11)], primarily in cortical regions, and have suggested that these result from changes in muscle afferent feedback either from direct action of BTX on afferents or secondary to changes in muscle activity itself. This may lead both to acute changes in brain function while the toxin is active and to longer term neural plasticity after changes in function are sustained for a period of time. Such an effect suggests BTX might help prevent spread or worsening of dystonic pathophysiology over time by reducing reinforcement of faulty motor programs, although the return of symptoms in most patients argues that BTX keeps pathophysiology at bay but is not able to eliminate it in most cases. We are unable to determine using DTI alone whether the changes observed in the current study reflect normal (function-related) white matter plasticity or if they reflect reversal of some pathologic process, such as inflammation, induced by aberrant neuronal activity.

How do these findings inform our understanding of the pathophysiology of dystonia? The basal ganglia circuitry is assumed to be a key player in at least some forms of dystonia (48–54). However, since the projections of this circuitry are small compared to major white matter tracts, it has been difficult to design imaging studies of dystonia that show direct evidence for abnormalities in basal ganglia circuitry, and as a result we have little empirical, data-driven information, other than observations with deep brain stimulation, on which to base our current hypotheses about the role of this circuitry in primary dystonias. The main goal of the current study was to address this challenge by using methods that maximized detection of signal from this circuitry in two ways. First, we used ROIs scaled (small, overlapping the region where pallidofugal projections are most dense) and placed (in the region colocalizing with the ansa lenticularis projection of the GPi) specifically in regions that would maximize the FA signal contributed by pallidofugal fibers. Second, repeated measures studies targeting treatment response are able to detect circuitry relevant to dystonia symptom expression better than cross sectional or oral medication studies because the intervention directly targets symptomatic muscles only. As a result of these two factors, we were able to detect FA changes that likely would not be observed using voxelwise comparisons or larger ROIs.

Having detected abnormalities and changes that we believe may localize to pallidofugal fibers, we took two additional steps to interpret our data. The first was to pose the question, what about the pallidofugal fibers might cause such an asymmetry in and/or a lateralized reduction in FA, and would this inform our understanding of dystonia? This first question was intended to be hypothesis-generating to drive future studies, rather than hypothesis-testing. Imaging is particularly well-suited for hypothesis generation due to its ability to capture new systems-level information in living human brains. Although such information may lack precise etiological specificity, it may narrow down possibilities to several potential interpretations that can be tested using other methodology (e.g., animal model or post mortem tissue studies). The second step was to conduct *post-hoc* tests to test whether our observations did indeed relate to pathophysiology, as opposed to mechanical compensation for head position. The results from these two steps are discussed below.

### What About the Pallidofugal Fibers Might Cause Such an Asymmetry (or Lateralized Finding), and Would This Inform Our Understanding of Dystonia?

While identification of abnormalities in the basal ganglia circuitry in this study is, to some degree, a methodological and proof-of-concept exercise, questions about the nature of the findings are more valuable in advancing our understanding of dystonia from a hypothesis-generating perspective. Since so little is still known about the role the basal ganglia play in dystonia pathophysiology, we hoped that whatever we detected in this region would allow us to raise new hypotheses to be tested in future studies.

When we had only preliminary evidence for the findings we have now tested more rigorously here (12), we asked what abnormalities and/or changes in pallidofugal fibers might lead to altered FA in this region. There are two primary factors that influence FA: The first is white matter density or myelination and the second is white matter coherence. The pallidofugal fibers traverse a robust bundle of cortipospinal projections, and likely contribute minimally to the FA signal in this region in a healthy brain (i.e., the descending fibers are the dominant factor in determining FA in this region). However, if these fibers were to be altered in certain ways (e.g., increased in density, or if their own architecture were to be less coherent), their signal might contribute in a more substantial way to FA in this region. Since increases in the density of these fibers would reduce coherence of fibers in the region, leading to less dominance of descending fibers, one would expect such an effect to lead to reduced FA. Reduced coherence or axon pathology in the pallidofugal fibers themselves might also lead to reduced FA in this region. So it is possible that in dystonia there are increased numbers of pallidofugal axons in the left hemisphere. But this did not completely satisfy us because it seemed unlikely that there would be changes in numbers of parent axons/cell bodies in this region with treatment. So we next searched the literature on pallidal output fiber anatomy and found that these axons not only collateralize but that there are two main motor fiber types, with one collateralizing substantially more than the other (55). Since more collateralization would lead to less coherence it seemed possible that the highly collateralized fibers could change the FA signal by increasing or decreasing the degree of collateralization or by simply exhibiting some type of reversible, function-related pathology, such as beading (56), that increased their contribution to the FA signal in this region before treatment and normalized with treatment. This literature on pallidofugal fiber anatomy led us to the idea that these two fiber types may correspond to functionally distinct systems within the basal ganglia. More specifically, the findings in the current study suggested the more diffusely collateralizing fiber type might point us to the functional system impacted in dystonia.

While the above ideas are complex and might be deemed overly speculative, the potential link between pallidofugal architecture and the pathophysiology of dystonia raises a concrete question that can be posed of known anatomical architecture in relation to dystonia: are there abnormalities in the number, collateralization, and integrity of the highly collateralizing fiber type in cervical dystonia patients, especially in the left hemisphere? This hypothesis was the key feature that defined the neural component of a novel conceptual model for dystonia that is able to explain a number of features of dystonia that have not previously been understood. Specifically, the model proposes that, dystonia reflects abnormal amplification of brain posture/stabilization function (44), and that the highly collateralizing fibers are central to gating and coordinating a mechanical impedance system that uses muscle co-activation and co-contraction to implement any number of functions that stabilize the body, including control of posture, balance, and fine-motor control. A full discussion of this model and associated hypotheses is detailed elsewhere (44, 45) and is outside the scope of the discussion here. While the testing of this model is in its infancy, a number of features of the model converge with existing literature. For example, neural control of posture appears to be left-lateralized, and this may explain the laterality of our findings here.

### Do Our Observations Relate to Pathophysiology and/or Compensation for Dystonia?

While the relationship of our findings to treatment effectiveness suggests our findings relate to dystonia pathophysiology, symptom reduction often goes hand in hand with reductions in other factors such as mechanical compensation for symptoms. While our study was not specifically designed to dissociate pathophysiology from compensation, we took advantage of the fact that there were dissociations between head position improvement, FA changes, and perception of treatment benefit in some patients after treatment. Such results should be seen as preliminary evidence to drive future studies. The results suggested that the FA abnormality and changes with treatment do not simply reflect changes relating to the degree of effort required to correct or compensate for altered head position, since correlations with these measures were weaker than for total dystonic severity in individuals who showed position improvement.

There were also individuals who showed corrected head position after treatment but did not feel that they received any benefit from the treatment. This dissociation allowed us to run preliminary analyses testing whether more psychological components of adaptation (distinct from pathophysiology) might relate to our findings, but there were no group differences in FA changes between individuals who did vs. did not feel benefit. Altogether, these findings suggest that the central changes are unlikely to reflect compensation only (i.e., they at least partly reflect changes in pathophysiology). However, since perception of benefit did not show any significant impact on FA measures or change with treatment, this suggests that the FA changes we see with treatment reflect changes in the expression of symptoms themselves, but may not identify or localize to the primary “driver” of the dystonia (i.e., the region encoding the affected motor program). Patients who do not feel benefit may be perceiving this “driver,” even if symptoms themselves are reduced. These latter points are consistent with the fact that symptoms nearly always return to some degree at the end of a treatment period; very rarely do patients receive injections and experience complete remission of dystonia.

One feature of the model we have proposed for dystonia is that the basal ganglia are involved in gating the programs affected in dystonia (and thus are involved in expression) but that the faulty programs themselves may be located in other motor regions such as the cerebellum, motor cortices, or brainstem motor nuclei. This would suggest that the “driver” of the dystonia would be in the motor region where the faulty program was located, but that the basal ganglia would affect when and how much that program was implemented. This does not, however, exclude the possibility that in some cases the “driver” could be the basal ganglia itself. These possibilities can be tested in larger cohorts looking at broader areas of the sensorimotor circuitry.

At this time it is not clear whether the relative left/right asymmetry or the left lateralized abnormality alone relates to dystonic symptoms and/or response to treatment, since FA abnormalities as a group were only observed in the left hemisphere, but some degree of change was associated with degree of improvement in each hemisphere (increasing in left and decreasing in right). However, we do not believe the answer to this question is critical to interpreting our findings because we do not believe it is an imbalance between the hemispheres itself that produces dystonia. Instead we believe the asymmetry reflects laterality in anatomical architecture and potentially of the different subsystems we propose are involved in left vs. right lateralized dystonia symptoms [(44, 45), also see next paragraph]. So this study leads to hypotheses that can be tested in future studies by selecting patients with specific qualitative features in their dystonia.

Because of the laterality of our findings, we ran *post-hoc* evaluations of treatment response of patients with predominantly left vs. right symptoms, using the net direction of head tilt as the determining factor. While we did not observe any direct relationship between asymmetry and symptom laterality, patients with right lateralized symptoms reported feeling less benefit from treatment than those with left lateralized symptoms. These clinical differences were not associated with any differences in FA asymmetry or changes, however. We can only guess at the reason for this but it may reflect differences in motor control programs on the dominant vs. non-dominant side of the body. For example, there might be differences in the extent to which these programs are feedforward vs. feedback-controlled, and since BTX exerts central effects through feedback mechanisms (motor afferents) one would assume that programs mediated by feedback would respond more favorably than those that are feedforward.

### Relevance of Our Findings to the Study of Adult White Matter Plasticity

In addition to the relevance of our findings to dystonia, the current findings contribute to the field of adult white matter plasticity. There has been increasing interest in and converging evidence for experience-driven white matter plasticity in adults [e.g., reviewed in (32, 37, 57–59)]. There is a growing body of animal literature evaluating adult white matter plasticity at the cellular/molecular level. This includes evidence for experience-driven hypertrophy of white matter astrocytic processes (33) and routine production of myelin-forming oligodendrocytes (30, 60) in adult rodents. In parallel, at least two studies have shown evidence for continuation of axon myelination into adulthood in rodents (61, 62). There is also preliminary evidence consistent with direct links between skill learning in adult rodents and changes in myelination as shown by two recent studies (34, 35). Interestingly, astrocytic processes appear to contribute approximately the same proportion of tissue as myelin in a standard-sized imaging voxel, so they are a strong candidate to consider when white matter changes/differences are observed in imaging studies (36). Astrocytic processes influence function and synchrony of membrane potential transmission and, along with oligodendrocytes, are potential candidates for modulating neural synchrony (63), which may be altered in a number of neurological and psychiatric disorders [e.g., (64–70)]. Previous work has also suggested a potential role for glia in the pathobiology of DYT1 dystonia (71); the findings here suggest additional, albeit indirect, evidence for a potential role of these neuromodulatory elements in dystonia.

In addition to animal literature, there have been a number of DTI studies in the human imaging literature showing evidence for adult white matter plasticity since our preliminary report in 2006 (12). Interventions in these studies associated with white matter microstructure changes have included training in or learning cognitive tasks (22–24), learning complex motor skills (25, 26), and administering treatment directly targeting the CNS (28). The BTX paradigm is an interesting complement to the other motor interventions in that one might argue the results here show motor “unlearning” (of a maladaptive motor program), rather than “learning,” suggesting that white matter changes can be seen in either direction.

### Study Limitations

As with any single research method and study, the findings come along with certain limitations. Below we itemize these and discuss how we minimized limitations in this study, or how they could be addressed in future studies.

1. All imaging studies face the conundrum that they rely on correlations with clinical features that are also likely themselves intricately correlated with other factors such as compensation for these features. That is, the worse the pathophysiology, the worse the symptoms and the more compensation for symptoms is needed. Thus, we cannot be certain that changes observed here relate directly to pathophysiology, as opposed to resulting from reductions in compensatory function (e.g., effort required to perform daily tasks) in conjunction with reduced dystonia symptoms. Fortunately, we were able here to take advantage of the fact that FA changes, positional correction, and patient perception that dystonia benefitted from the injections did not align precisely with each other, and ran analyses that provide preliminary evidence that the findings reflect pathophysiology related to expression of dystonia. Future studies should use more direct measures of disability, such as the disability and pain scores in the dystonia scales, to test this hypothesis more directly.

2. Our inclusion of individuals with little or no previous exposure to BTX in the current study meant we could not predict the strength of response to BTX for these individuals, or rely on established BTX regimens with known beneficial outcome. Responses to BTX injections vary substantially across individuals, ranging from no benefit to near complete remission (72) and depend on a number of patient, disease, and physician variables. The inability to predict response strength, and resulting low or absent changes in scale scores, in some study participants suggests that our power to detect treatment-related FA changes was probably not optimized in the current study. At the same time, the range in symptom improvement was a strength of study design because it allowed us to assess the relationship between FA asymmetry change and clinical improvement, and also to dissociate positional improvement from perceived clinical benefit. In the future, studies are needed to assess FA changes in larger cohorts of individuals with an established, robust response to BTX. In addition, as mentioned in the methods section, it is important to note that dystonia scales may not always be the most accurate measure of the degree to which symptoms have improved, particularly in patients whose injection patterns have not yet been optimized.

3. Sample size: The sample size of this study was modest, but not substantially different from a number of previous dystonia publications. In addition, a number of factors yield the sample size here less of a limitation than in some other studies: (a) repeated measures studies usually yield greater statistical power for a given cohort size than cross sectional studies [73] since each subject serves as its own control. This is particularly true when the variable being measured is relatively stable in an individual over time in the absence of intervention; thus, data from structural imaging modalities (e.g., data from mprage and DTI sequences) should be more stable than from functional modalities (e.g., fMRI and resting state MRI). (b) Our study was driven by *a priori* hypotheses, limiting the number of comparisons made and predicting the direction and nature of change with treatment. This reduced the likelihood of false positives substantially, compared with similar size cohort studies using whole brain voxelwise approaches to analysis. (c) We related imaging findings to behavioral/clinical differences to gain greater confidence that the changes were treatment-relevant [e.g., (22, 26, 28)]. (d) The study also included a hypothesis-generating component that can be tested more directly with other methods. That is, the statistical power of the current study needed to be strong enough to motivate future studies, but was not essential to confirming the generated hypotheses.

4. Use of two MRI scanners in this study: it is unfortunate that two different MRI scanners were used for a relatively small cohort in this study. We used the following approach to minimize any effect(s) that this factor imposed on the data: (1) we used a repeated measures design wherein the same scanner was used across sessions for all subjects; thus between session changes were relative, rather than absolute measures. (2) All demographically matched patient control pairs were scanned on the same magnet so the same number of patients and controls were scanned on each magnet, and demographic factors were matched one-to-one across magnets. (3) The primary measure of interest was a relative measure (left/right asymmetry) of FA, minimizing any baseline differences in scanner calibration.

5. Interpreting findings using general microstructural markers (FA, MD, AD, RD): Since the imaging modality and specific markers evaluated here were fairly general measures that are not specific to a particular pathology or etiology, future studies are needed to test questions about biological interpretation more directly. One approach would be to use MR sequences specialized for detecting specific etiologies (e.g., T2^*^ to evaluate myelination or iron deposition). However, if the main difference between patients and controls arises from architectural features of the neurons in this region rather than pathology, such sequences may not provide any specific insight. Instead, we suggest that our findings should direct us immediately to a methodology that can more directly evaluate the tissue in this region, such as post mortem studies of the two subtypes of motor pallidal output projections.

## Summary

A left/right asymmetry in brain white matter microstructure medial to the GPi in CD was reduced 4 weeks after peripheral BTX injections. There was a linear relationship between the magnitude of white matter changes and clinical response to treatment; this relationship was diminished when evaluating factors relating to compensation alone (distinct from pathophysiology). These findings provide evidence that BTX treatment in dystonia affects function of the basal ganglia circuitry, and support a role for this circuitry in the expression of cervical dystonia symptoms. The laterality of the asymmetry is consistent with a conceptual model that proposes dystonia reflects amplification of a left-lateralized functional brain system, namely the system controlling posture and body stabilization.

## Ethics Statement

This study was carried out in accordance with the recommendations of the Institutional Review Board (IRB) of Partners HealthCare (the Partners Human Research Committee) with written informed consent from all subjects. All subjects gave written informed consent in accordance with the Declaration of Helsinki, and all experiments were HIPAA compliant. The protocol was approved by the Partners Human Research Committee.

## Author Contributions

AB: conception and design of the work, acquisition, analysis, and interpretation of data for the work, drafting of the manuscript and revising it for important intellectual content, accountable for all aspects of the work in ensuring that questions related to the accuracy or integrity of any part of the work are appropriately investigated and resolved. JK and JL: acquisition, analysis, and interpretation of data for the work, revising manuscript for important intellectual content. JW and NS: acquisition and analysis of the work, revising manuscript for important intellectual content. TM-B: acquisition and analysis of the work, revising manuscript for important intellectual content. LS: acquisition of the work, revising manuscript for important intellectual content. HB: Interpretation of the data for the work, revising manuscript for important intellectual content.

### Conflict of Interest Statement

The authors declare that the research was conducted in the absence of any commercial or financial relationships that could be construed as a potential conflict of interest.

## Supplementary Material

Figure S1 Relationship of FA asymmetry changes to clinical improvement in CD using the TWSTRS scale. We computed this correlation only for patients who experienced a net improvement in their scale scores (7 of 11 patients with TWSTRS scales at both scanning sessions; see Methods).

Figure S2 Relationship of individual hemispheric FA changes to clinical improvement in CD using Tsui dystonia scale severity measures. **(A)** Relationship of left hemisphere FA changes with treatment to reduction in symptom severity. **(B)** Relationship of right hemisphere FA changes with treatment to reduction in symptom severity.

Figure S3 Relationship of FA hemispheric asymmetries in CD to symptom severity using the TWSTRS scale at the pre-injection (“before BTX") scan (*n* = 12 patients who had pre-injection TWSTRS scores).

Figure S4 Relationship between postural effort required to counteract head deviation (distinct from total severity of dystonia) and FA measures, before treatment [**(A)** FA asymmetry, **(B)** left hemisphere FA, and **(C)** right hemisphere FA] for all patients.

Figure S5 Relationship between percent reduction in postural effort values and changes in FA measures, after treatment [**(A)** FA asymmetry, **(B)** left hemisphere FA, and **(C)** right hemisphere FA] for all patients.

Table S1 Supplementary Methods.

Data Sheet S1 Raw Clinical Data.

Data Sheet S2 Raw data extracted from fractional anisotrphy (FA) maps using region of interest masks.

Data Sheet S3 Raw data extracted from mean diffusivity (MD) maps using region of interest masks.

Data Sheet S4 Raw data extracted from the second and third eigenvalue maps (L2 and L3) using region of interest masks, to compute radial diffusivity (RD).

Data Sheet S5 Raw data extracted from the first eigenvalue map (L1) using region of interest masks, to compute axial diffusivity (AD).
