# Supplementary figures and images for: White Matter Changes in Cervical Dystonia Relate to Clinical Effectiveness of Botulinum Toxin Treatment
Source: Front Neurol. 2019 Apr 4;10:265. doi: 10.3389/fneur.2019.00265 (PMC6459077; doi:10.3389/fneur.2019.00265)

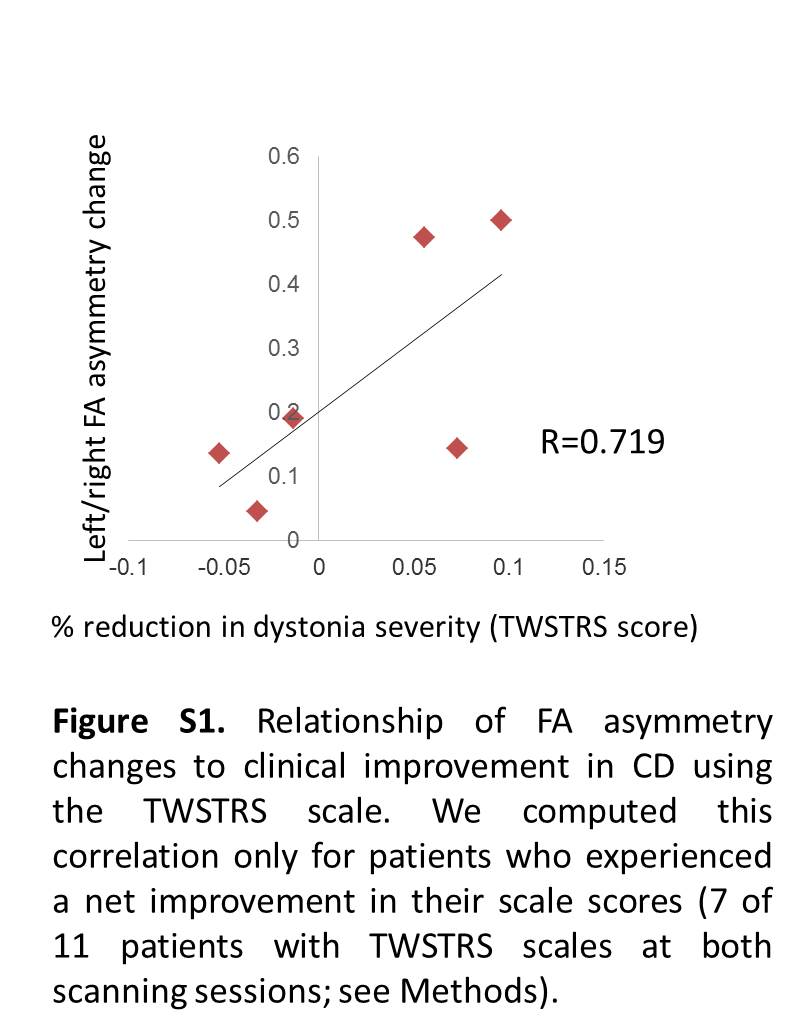

Supplement: Figure S1 — Relationship of FA asymmetry changes to clinical improvement in CD using the TWSTRS scale. We computed this correlation only for patients who experienced a net improvement in their scale scores (7 of 11 patients with TWSTRS scales at both scanning sessions; see Methods). [file Image_1.jpg]

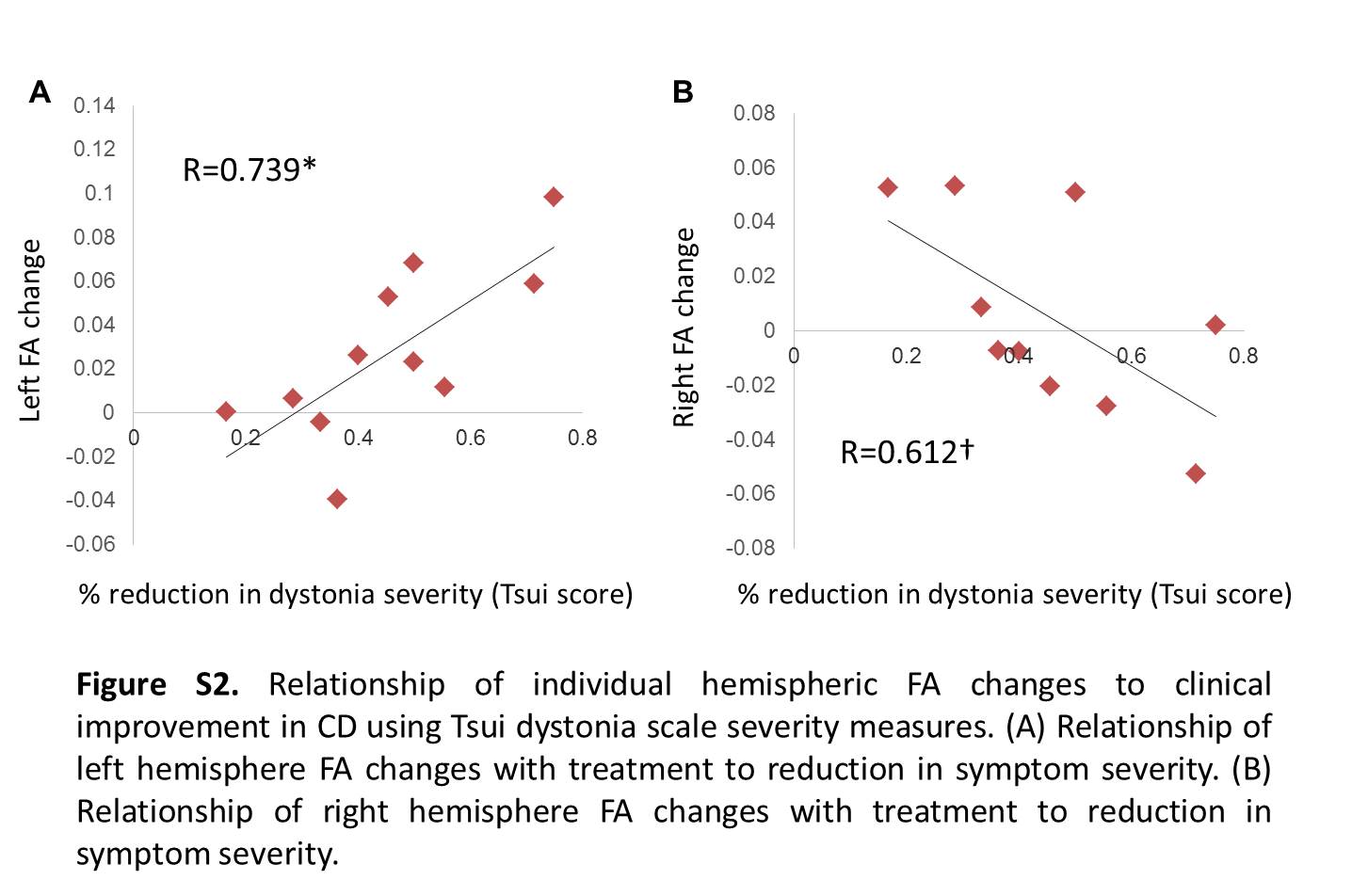

Supplement: Figure S2 — Relationship of individual hemispheric FA changes to clinical improvement in CD using Tsui dystonia scale severity measures. (A) Relationship of left hemisphere FA changes with treatment to reduction in symptom severity. (B) Relationship of right hemisphere FA changes with treatment to reduction in symptom severity. [file Image_2.jpg]

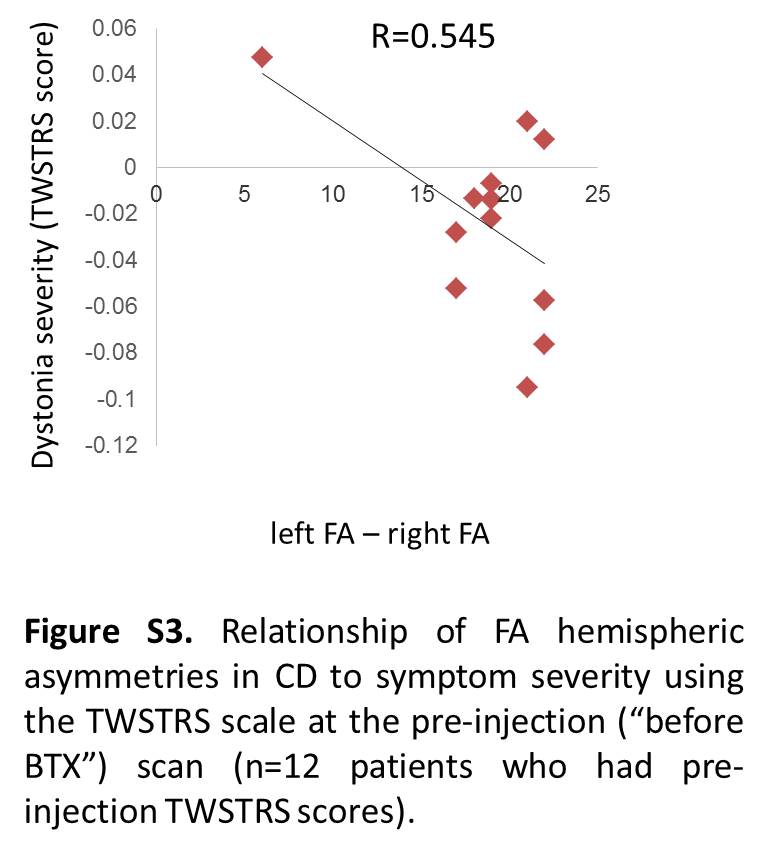

Supplement: Figure S3 — Relationship of FA hemispheric asymmetries in CD to symptom severity using the TWSTRS scale at the pre-injection (“before BTX") scan (n = 12 patients who had pre-injection TWSTRS scores). [file Image_3.jpg]

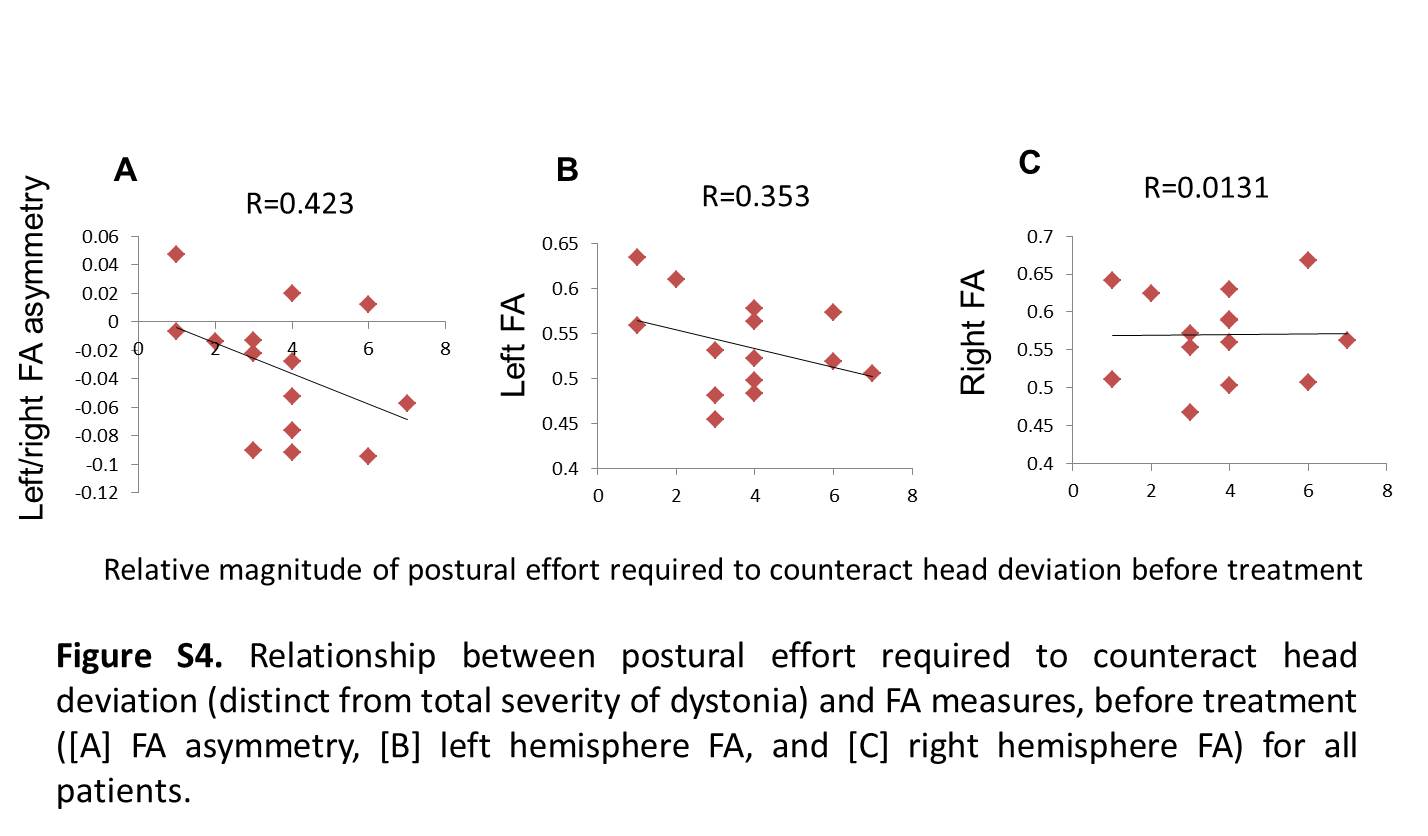

Supplement: Figure S4 — Relationship between postural effort required to counteract head deviation (distinct from total severity of dystonia) and FA measures, before treatment [(A) FA asymmetry, (B) left hemisphere FA, and (C) right hemisphere FA] for all patients. [file Image_4.jpg]

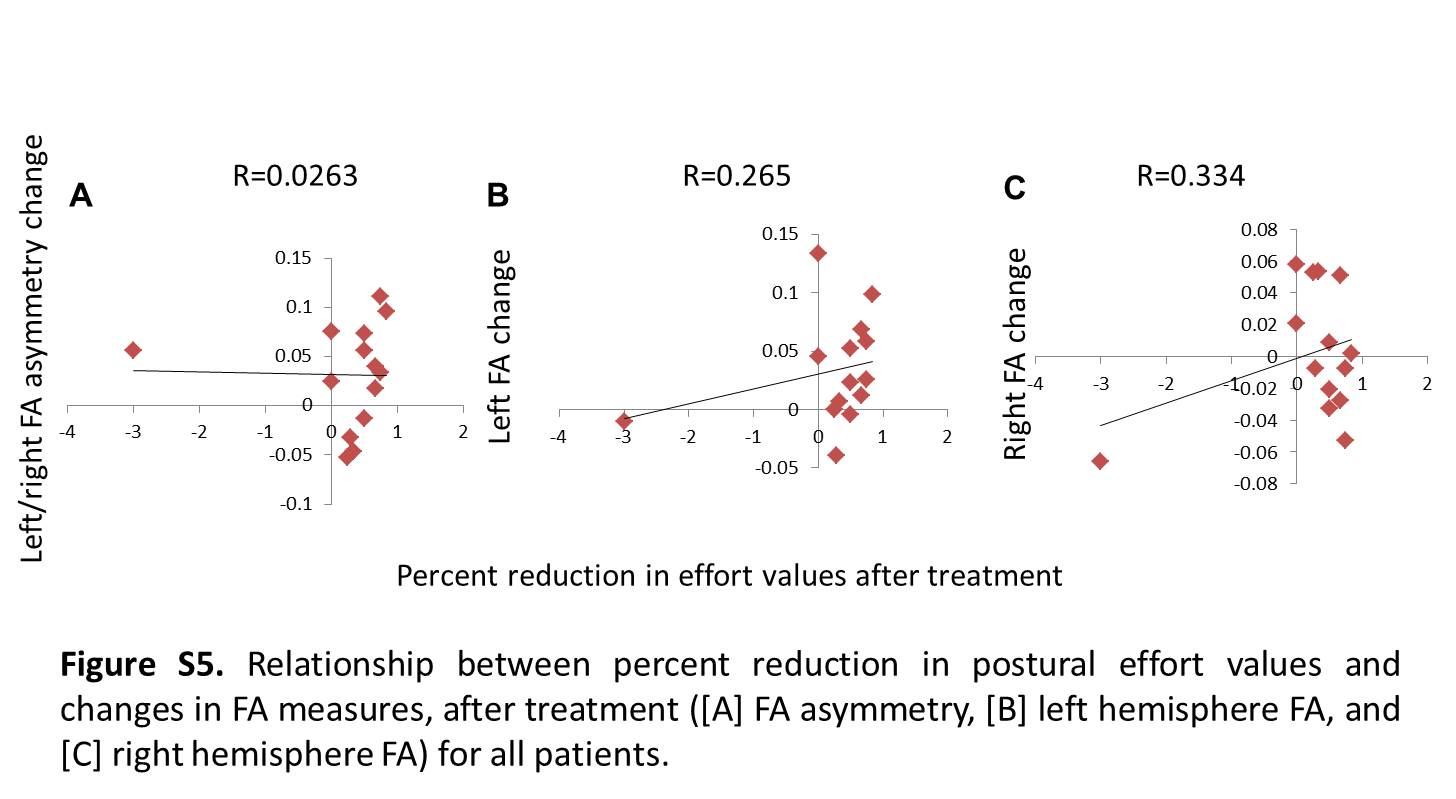

Supplement: Figure S5 — Relationship between percent reduction in postural effort values and changes in FA measures, after treatment [(A) FA asymmetry, (B) left hemisphere FA, and (C) right hemisphere FA] for all patients. [file Image_5.jpg]
