# Supplementary material for: White Matter Changes in Cervical Dystonia Relate to Clinical Effectiveness of Botulinum Toxin Treatment
Source: Front Neurol. 2019 Apr 4;10:265. doi: 10.3389/fneur.2019.00265 (PMC6459077; doi:10.3389/fneur.2019.00265)
Supplement: Table S1 — Supplementary Methods. [file Table_1.DOCX]

**Supplementary Methods**

**STANDARDIZED motor exam protocol**

15-30 minutes for completion

**Preparation**:

Ask subject to - 1) Roll-up sleeves to elbow & pants to the knee 2) Remove shoes, socks, coats, scarves, hats, glasses, etc.

3) Tie up/back long hair

NOTE: Conduct protocol with subject sitting directly opposite you.

**WHOLE BODY**:

- Ask subject to sit in a chair (preferably a chair without arms and 6” from wall) at rest, feet flat on the floor, arms relaxed with hands sitting on each thigh. Film whole body for 10 seconds.
- Observe lower body, feet, upper body and face. While observing chest & face ask the subject to state the months of the year starting from December going backwards.

**HEAD**: (Focus on chest & face)

Ask subject to:

- Look straight ahead; relax and let your body do what it wants. Close your eyes for about five seconds.
- Look straight ahead and try to hold your head in a neutral position.
- Slowly turn head to the left, to the right, up, straight ahead, and downward.
- Look straight ahead, slowly tilt head to the right, touching ear to shoulder. Repeat to the left
- Demonstrate any sensory tricks

**FACE**: (Focus on face & neck)

Ask subject to:

- Open and close your eyes tightly five times.
- Open and close your jaw five times
- Open your mouth, stick out your tongue, move your tongue side to side; close your mouth

**ARMS AND HANDS**: (Focus on upper part of body)

Ask subject to:

- Place arms outstretched straight in front of you, with fingers spread out, and palms down. Close your eyes
- Keep your eyes closed. Slowly turn hands over so that your palms face the ceiling.
- Slowly turn palms down again. Open your eyes.
- First with your right hand, turn your palm to the ceiling then the floor as full and as fast as possible 10 times.
- Now the same with your left hand.
- Make a “wing” position: (both elbows straight out to the side, each elbow bent with palms of hands facing the floor, and both hands facing toward each other; close but not touching)
- Close your eyes and hold 10 seconds

**RAPID SUCCESSIVE MOVEMENTS**: (Continue to focus on chest & head with focus on hands)

Ask subject to:

- First on the right, make big, quick taps with your first finger and thumbs until I say stop.
- Now the same on the left.
- First on the right, fully open and close fists as though flinging water until I say stop.
- Now the same on the left.

Switching to your feet: (CHANGE focus to the feet)

- Stomp foot on the floor until I say stop, lifting entire foot off the floor
- First on the right, tap toes until I say stop keeping heel on the floor
- Tap your heel until I say stop, keeping your toes on the floor
- Tap heel-out / toe-back until I say stop, alternating heel and ball of foot
- Now the same on the left.

**FINGER-TO-NOSE**: (Focus on chest, head, arm, elbow and finger)

*Hold your finger out in front of the subject about an arm’s length away from the subject’s face; move your finger so subject reaches 2x in each position*

*Make sure that the subject is extending his/her arm FULLY when reaching to touch your finger*

Ask subject to:

- Use your index finger to touch your nose, then SLOWLY extend your arm to touch your finger
- Repeat 5 times with each hand.

**RIGIDITY**: (Focus on the full body)

Ask the subject to:

- Relax as much as possible

*Slowly test the range of motion and tone of neck, R/LUE, R/LLE; Start with the extremities and end with the neck. With each maneuver, if the tone is normal and with full range of motion, ask the subject to open and close the contralateral hand.*

**VOICE**: (Focus on upper body)

Ask the subject to:

- Comment on/Talk about the weather to get a 1 minute voice sample.
- Ask whether s/he has swallow problems or choking
- Ask subject to clear his/her throat
- Take deep breath before each:
  - Hold a long “aaahhh” (as in CAT) sound for 10 seconds
  - Hold a long “eeeeee” sound for 10 seconds
- Please read the following sentences one at a time, as if you were speaking to

somebody in a real conversation (attached below).

Read the “Rainbow Passage” and sentences (attached below)

**STANDING**: (Focus on the full body – head to toe at full height)

Ask subject to:

- Cross your arms over your chest and stand up unassisted; uncross arms
- Face the clinician, then make 4 quarter turns, stopping after each turn

**PULL TEST**: (Continue head-to-toe focus)

*Be prepared to catch that subject if he/she falls. Stand with your back about 2 feet from a wall.*

Ask subject to:

- Stand relaxed, with feet spread slightly apart.

*Stand about 2 feet behind the subject. Place your hands on the subject’s shoulders and gently shake. Then quickly but gently pull back on the subjects shoulders, allowing the subject to catch his/her balance.*

*Repeat 2-3 times.*

**WALKING**: (Continue head-to-toe focus)

Ask subject to:

- Walk up and down long hallway, going back and forth three times.

(switch focus on lower body)

- Walk toward me heel-toe, as if you’re on a tight rope.
- Walk backwards normally for 15 steps.
- Walk forward on your tip toes for 10 steps.
- Walk forward on your heels for 10 steps.

**WRITING**: (Focus on the full body but also down the arm and on the upper body, then on hands)

Ask the subject to WRITE:

- ‘Today is a sunny day in southern California’ three times with his/her dominant hand
- A line of connected cursive L’s/loops from one side of the page to the other; first with dominant, then non-dominant hand
- Create a spiral; start small in the middle of the page and get progressively larger; make sure to keep elbow and wrist off table; first with dominant, then non-dominant hand

**While completing motor/movement scales**:

- Observe whole body for resting tremors (focus on face, lips, chin, upper extremities, and lower extremities).
- Observe head, face and walking for neck rotation, flexion, extension and shoulder elevation, anterior and posterior displacement
- Observe the whole body, seated, standing and walking for upper and lower trunk rotation, lateral deviation, flexion or extension
- Observe whole body, tapping and walking for lower extremity deviations, then feet flat on the floor.

THE RAINBOW PASSAGE

When the sunlight strikes raindrops in the air, they act like a prism and form a rainbow. The rainbow is a division of white light into many beautiful colors. These take the shape of a long round arch, with its path high above and its two ends apparently beyond the horizon. There is, according to legend, a boiling pot of gold at one end. People look, but no one ever finds it. When a man looks for something beyond his reach, his friends say he is looking for the pot of gold at the end of the rainbow.

Fairbanks, G. (1960) Voice and Articulation Drillbook, New York, Evanston, and London: Harper & Row

The blue spot is on the key again.

How hard did he hit him?

We were away a year ago.

We eat eggs every Easter.

My mama makes lemon jam.

Peter will keep at the peak.

Subject ID Number: __________________

Date: __________________

1. Please write the following sentence three times:

Today is a sunny day in southern California.

1. Please make a series of connected loops (cursive lower case L’s) across the entire width of the page.

-- -- -- -- -- -- -- -- -- -- -- -- -- -- -- -- -- -- -- -- -- -- -- -- -- -- -- -- -- -- -- -- -- -- -- -- --

1. Please make a spiral that fills the space below. Please hold the pen so that your wrist and elbow are off the table.
